# Addressing culture within healthcare settings: the limits of cultural competence and the power of humility

**Published:** 2019-03-13

**Authors:** Lauren MacKenzie, Andrew Hatala

**Affiliations:** 1 Department of Community Health Sciences, Max Rady College of Medicine, University of Manitoba, Manitoba, Canada; 2 Section of Infectious Diseases, Department of Internal Medicine, Max Rady College of Medicine, University of Manitoba, Manitoba, Canada

## Introduction

For many years, social scientists have been stressing the important role culture plays in the work of healthcare providers in their clinical settings.^1^^,^^2^ This idea now saturates official statements and guidelines published by professional medical associations. In an article outlining the duties and responsibilities of physicians in Canada, for instance, the Canadian Medical Protective Association describes the importance of “provid[ing] culturally competent and culturally safe care.”^3^ Detailing the abilities and roles of a “competent” physician, the Royal College of Physicians and Surgeons of Canada CanMEDS framework similarly focuses on the delivery of patient-centered care, which involves understanding a patient’s values, beliefs, biases, and family dynamics.^4^ Appeals for enhanced “cultural competence” also abound from researchers and physicians working to improve Canadian Indigenous health.^5^ Together, such appeals and guidelines direct attention towards aspects of a patient’s culture that must be understood in order to effectively address his or her healthcare needs.

### Limitations of cultural competency

While many definitions of culture exist in the literature, a recent Lancet Commission proposed that culture “can be thought of as a set of practices and behaviours defined by customs, habits, language, and geography that groups of individuals share.”^2^ As healthcare providers and educators, we are becoming increasingly familiar with the intersection between culture and health that has led to positive outcomes in terms of promoting diversity, inclusion, and respect within clinical practice.^2^^,^^6^ There are nevertheless important shortcomings within these perspectives—and with notions of cultural competence in particular—that should be discussed in order to help us build a stronger, more integrated approach to addressing culture within healthcare settings.

The central problem with the concept of cultural competence is its focus on the “other.” Indeed, as a recent letter in CMAJ argued, “it is important that current medical training includes and gives due importance to training culturally competent doctors,” which involves “requiring appropriate knowledge about different cultures, cultural norms and cultural values.”^5^ This view of cultural competence implies that a cultural outsider (i.e., healthcare professional) can achieve a level of knowledge adequate to “know” a particular culture—to become competent.^6^ Framed in this way, however, culture can be reduced inadvertently to a simplified and static “tick box” of sorts - an item to be checked-off a medical training to-do list.^1^^,^^2^^,^^7^ This is particularly relevant given the current shift towards competency-based medical education.^4^ Yet, by distinguishing “cultural” competence from a more general “medical” competence, we further imply that “cultural” and “medical” are mutually exclusive, when in fact the two are intimately connected.^6^

We agree that in Canada “there is an urgent need to explore specific evidence about which strategies are most effective for improving both culturally competent healthcare and the delivery of culturally safe clinical care to First Nations communities.”^4^ However, by emphasizing the achievement of cultural competence, we risk glossing over the heterogeneity and fluidity inherent within culture(s).^1^^,^^2^ For example, what it means to be Indigenous or First Nations is not the same thing to all individuals who identify as such; nor is such an identity impervious to change over time or through contact with new ideas or ways of life. By focusing on building competence of the “other,” the “cultural” within cultural competence can inadvertently become synonymous with a static vision of race, ethnicity, or nationality, thereby unintentionally reinforcing problematic stereotypes.^6^ Moreover, other authors note that this notion of cultural competence can also overlook power imbalances and reinforce processes of marginalization that helped to create this “othering,” and assert at the extreme end that “cultural competency promotes an obsolete view of culture and is a form of new racism.”^7^

### The cultures of biomedicine

A more comprehensive strategy for educational initiatives aimed at improving clinicians’ understanding around the role of culture within healthcare settings must involve critical self-reflection on the culture(s) of biomedicine. How are we as clinicians being trained to see the world and how might that vision impact our clinical care?

The cultures of biomedicine are painstakingly learned and assimilated during many years of medical education and residency.^1^^,^^2^^,6^ Contrasting physicians’ current versus before-medical-school selves, it becomes readily apparent that a new culture is assumed—a new way of writing, speaking, of seeing the human body, of hearing and interpreting illness narratives, and even navigating a new political and power hierarchy.^1^ Viewing the world through this biomedical lens can also involve assumptions and biases about mind-body dualism, rigid power hierarchies, and forms of reductionism, individualism, or materialism that can translate into unnecessary hospitalizations, overreliance on prescription medications, or excessive diagnostic testing.^2^ Given that healthcare is also a cultural system with its own specific language, values, and practices, it is important to reflect on the challenge faced by patients and their family members as they translate, interpret, and negotiate through it.^2^^,^^8^

Current appeals for enhanced cultural competence within clinical settings lack a full appreciation for the influence of biomedical cultures—ways of seeing and habits of acting.^3^^-5^ This is not surprising given that “the hardest thing to know in a relative and comparative sense might be one’s own culture: what anthropologists call the anthropological paradox.”^2^ By recognizing the influence of biomedical cultures, however, important biases and values can be examined, critiqued, and taken into consideration. Can we as physicians become more aware of our own cultural perspectives so we do not bring unintended harm to others by intervening naively, excessively, or inappropriately (for example)?

### Humility over competence

This question leads us to the important notion of cultural humility. Rather than a focus on increasing “competence” and expert knowledge of the cultural “other,” cultural humility directs attention toward healthcare providers and clinical settings. First outlined in 1998, cultural humility has three dimensions: 1) lifelong learning and critical self-reflection; 2) recognizing and challenging power imbalances; and 3) institutional accountability.^9^ This critical self-reflection is more than self-awareness; it requires one to step back to understand one’s own systematic biases, values, and cultural assumptions, and the politics and institutionalized power dynamics latent within biomedical contexts. When it comes to understanding another’s experience, it involves a “way of being” with patients that values and humbly acknowledges oneself as a learner, relinquishing the role of competent “expert.”^9^ *In this way, subtle, and perhaps overt*, ideas of ethnocentrism and racism—where the underlying implication that a patient’s problem is due to “cultural” difference—are mitigated rather than reinforced.^7^^-^^9^

Although perhaps challenging to break through the “anthropological paradox,”^2^ current research on the practices of “mindfulness” in the clinic suggest that this humble and self-reflective “way of being” can be cultivated through noticing bodily cues, reflective attention to one’s thoughts and emotions, and an awareness and sensitivity to context, circumstances, and environment.^10^ In practice, Chang and colleagues have also shown that by following a cultural humility model, physicians can achieve improved patient satisfaction, better medical adherence, and enhanced health outcomes.^11^

This need for humility and critical reflection also applies to our healthcare systems and institutions. Although it may seem relevant to question how “to build health care institutions that are sustainably culturally competent throughout Canada,”^5^ this focus on “competence” is equally problematic when discussed at institutional levels. Instead, by teaching and fostering an organizational environment of critical self-reflection, we can be encouraged to recognize, critique, and challenge inherent biases within biomedical settings.^9^ Supported in this way, institutionalized cultural humility can contribute to: 1) non-paternalistic styles of communication and collaboration; 2) improved patient-centered care; 3) stronger patient rapport, cooperation, and partnership building; and 4) improved ratings of patient and hospital safety culture.^8^ In this way cultural humility helps pave the way for “culturally safe” clinical care;^12^ that is, healthcare built around respectful, open communication that acknowledges innate power differentials within the healthcare system.^12^ Unlike cultural competence, determining whether culturally safe care has been achieved is decided by those receiving (and not administering) healthcare, and is ultimately an outcome of institutionalized and cultural practices of humility in healthcare settings.^12^

### Conclusion

As Canada continues to become more diverse, healthcare providers will be encouraged to become more aware of cultural complexity and its impact on health.^7^^-^^9^^,^^11^ The adoption of cultural competence teaching within medical curricula was an important step. However, this approach does not fully capture the complexity and dynamism inherent to culture and fails to acknowledge the culture(s) of biomedicine we are situated in as care providers. Without recognizing the role of culture in biomedical practice, we cannot fully implement a patient-centered approach to care. Applying the concept of cultural humility, its critical self-reflection, and its institutionalization in culturally safe environments, is an important next step towards meaningfully addressing culture within healthcare settings.
